# A review of the pathophysiological mechanisms of doxorubicin-induced cardiotoxicity and aging

**DOI:** 10.1038/s41514-024-00135-7

**Published:** 2024-01-23

**Authors:** Annet Nicole Linders, Itamar Braga Dias, Teresa López Fernández, Carlo Gabriele Tocchetti, Nils Bomer, Peter Van der Meer

**Affiliations:** 1 Department of Cardiology, University Medical Center Groningen, University of Groningen, Hanzeplein 1, PO Box 30.001, Groningen, The Netherlands; 2 Division of Cardiology, Cardiac Imaging and Cardio-Oncology Unit, La Paz University Hospital, IdiPAZ Research Institute, Madrid, Spain; 3 Department of Translational Medical Sciences (DISMET), Federico II University, Naples, Italy; 4 Centre for Basic and Clinical Immunology Research (CISI), Federico II University, Naples, Italy; 5 Interdepartmental Centre of Clinical and Translational Sciences (CIRCET), Federico II University, Naples, Italy; 6 Interdepartmental Hypertension Research Centre (CIRIAPA), Federico II University, Naples, Italy

**Keywords:** Cardiovascular diseases, Senescence

## Abstract

The population of cancer survivors is rapidly increasing due to improving healthcare. However, cancer therapies often have long-term side effects. One example is cancer therapy-related cardiac dysfunction (CTRCD) caused by doxorubicin: up to 9% of the cancer patients treated with this drug develop heart failure at a later stage. In recent years, doxorubicin-induced cardiotoxicity has been associated with an accelerated aging phenotype and cellular senescence in the heart. In this review we explain the evidence of an accelerated aging phenotype in the doxorubicin-treated heart by comparing it to healthy aged hearts, and shed light on treatment strategies that are proposed in pre-clinical settings. We will discuss the accelerated aging phenotype and the impact it could have in the clinic and future research.

## Introduction

In recent years, the number of cancer survivors has increased. In Europe alone, 12 million patients were successfully treated for cancer^1^. Among this group, the long-term cardiac side-effects of cancer therapies are becoming evident. Between 1 and 5% of cancer survivors show signs of cancer therapy-related cardiac dysfunction (CTRCD), and 20% of these patients show asymptomatic LV function reduction^2,3^. In childhood cancer survivors who reached the age of 50, this percentage was even higher. The cumulative incidence of at least one symptom of cardiovascular disease was 45.5%, while this was only 15.7% in controls^4,5^. One of the chemotherapeutic drugs that is notorious for causing CTRCD, is doxorubicin^3^. Doxorubicin-induced cardiotoxicity (DCT) is defined by a decline in left ventricular ejection fraction of more than 10% to a value smaller than 53%^2,6^. This happens in up to 9% of the patient population treated with doxorubicin, depending on the received cumulative dose^7,8^.

The mechanisms underlying DCT are extensively investigated, but no conclusive evidence has been found. Proposed mechanisms include apoptosis, mitochondrial dysfunction, calcium dysregulation, inflammation, and oxidative stress^8,9^. One hypothesis that is supported by an increasing pool of evidence, is that doxorubicin causes a phenotype of accelerated aging, resulting to changes in the mentioned processes^10–14^. This is illustrated by studies in patients who were treated with doxorubicin during their childhood. These individuals show physical traits that are more similar to an older population, in terms of frailty, endurance, development of life-threatening diseases, and muscle strength^10,13,15^. Aging is also visible at the cellular level, where it is termed cellular senescence^14,16–18^.

## Doxorubicin: molecular and biological characteristics of a potent cancer treatment

Doxorubicin was discovered in 1969 as a homolog of daunorubicin, and was isolated from a soil bacterium, *Streptomyces peucetius*^8,19^. It is a widely used chemotherapeutic drug to treat solid tumors, leukemia, and lymphoma both in adults and children^6^. The classic antitumor effect of doxorubicin is most often attributed to the inhibition of Topoisomerase IIb (TOP2B). TOP2B is an enzyme that prevents double stranded nuclear DNA breaks (DSBs) caused by stresses in the strands. These can build up due to transcription or replication^20^. Besides this, doxorubicin is involved in many other mechanisms directly and indirectly underlying DSBs, such as increased reactive oxygen species (ROS) production and mitochondrial dysfunction^8^.

Implementation of doxorubicin in cancer treatment regimens has been very beneficial, as reflected by the reduced the risk of death in ovarian cancer by 15% (HR = 0.85 [CI 0.76–0.95], *p* < 0.001)^21^. In metastatic breast cancer, doxorubicin led to a survival advantage of 13% (HR = 1.13 [CI 1.00–1.27])^22^. However, by this time it was also evident that doxorubicin could cause cardiotoxicity in a dose dependent manner. In a large meta-analysis, 22,815 doxorubicin-treated patients, 17.9% of these patients developed sub-clinical cardiac dysfunction, while 6.3% of them developed clinical heart failure. Cumulative dose was reported as the most important predictor of DCT^23^.

Many mechanisms for DCT have been proposed^9^. However, controversy remains due to varying doses of doxorubicin used in preclinical experiments. In human blood, the concentration after doxorubicin administration is typically between 0.025 and 0.250 µmol/L^24–28^, whereas in vitro experiments often apply doses of 1 µmol/L or higher. Additionally, mice can tolerate much higher doxorubicin concentrations, with tolerable blood concentrations reaching 0.7–2.1 µmol/L (10–30 mg/kg)^29,30^. Doxorubicin treatment has shown different pharmacological effects at different concentrations: At sub-micromolar concentrations, the primary mechanism of action is on DSB formation, while this shifts to free radical toxicity at 2–4 µmol/L^19^. Therefore, the accelerated aging phenotype might have been overlooked in experimental setups assessing cardiomyopathy^31^.

## Phenotypic overlap between the aged and the doxorubicin-treated heart

As the heart ages, it undergoes many transformations, affecting morphology, electrophysiology gene expression, and metabolism, all of which have a substantial influence on cardiac output. Particularly, the heart of a patient with DCT exhibits more similarities with the heart of an aged individual than with that of healthy peers^10,13,15^. Both the aged and DCT heart show decreased atrial emptying rates, a measure of diastolic dysfunction^31–36^. This is linked to development of atrial fibrillation (AF)^37–39^ Incidence of AF increases with age: below the age of 55, 0.1–0.2% of the population has AF, while this number increases to 9.1–11.1% above 85 years^40,41^. In DCT patients (46 ± 15 years), the incidence of atrial fibrillation lies around 10%^38,39^. Additionally, both the aged and DCT treated heart show prolonged QT intervals, pointing at aberrant electrophysiology^38,42,43^.

On a morphological level, hypertrophy was observed in experimental models of both the aged^44–50^ and the DCT heart^51–54^, as well as in patients when measuring LV mass index^32,55,56^. When assessed microscopically, both the aged and DCT heart show accumulation of waste products, shown with lipofuscin staining and increased β-galactosidase activity^18,44,57–59^. Furthermore, increased fibrosis is observed (1.5x increase in the aged heart, 2.2x increase in the DCT heart^18,60^). In DCT mRNA expression of collagen fibers is not upregulated^61^, while protein levels are increased^62^. Besides, cardiac fibroblasts show increased expression of matrix metalloproteases (MMPs) and tissue inhibitors of MMPs (TIMPs), suggesting increased remodeling^50,60,61^. Inhibition of MMPs during doxorubicin treatment prevents DCT^63^.

Overall, there are marked similarities between aged and, often younger, DCT hearts, supporting the hypothesis that doxorubicin induces an accelerated aging phenotype.

## Molecular consequences: cellular senescence as a hallmark of the aging process

**Fig. 1:**
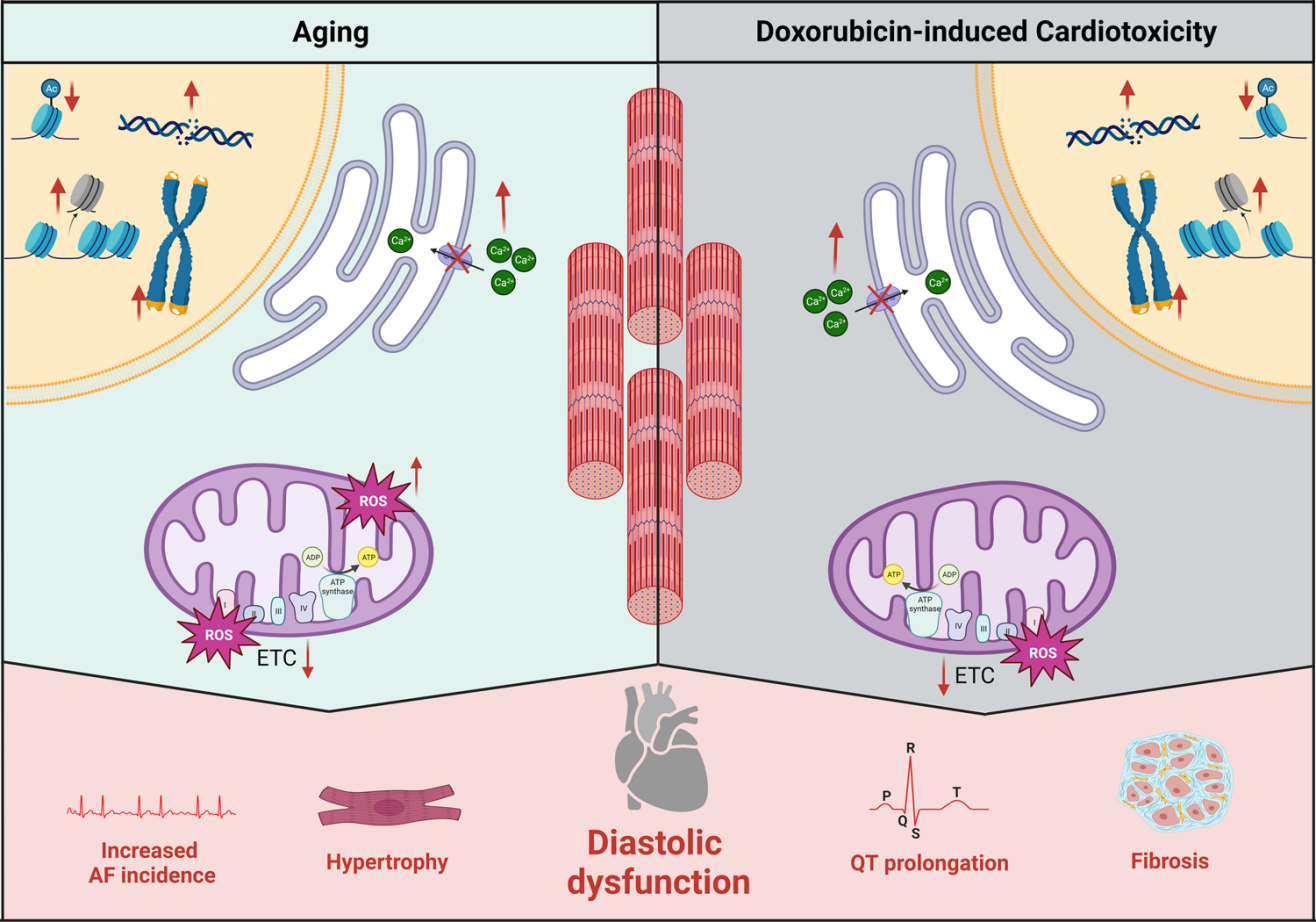
Overview of mechanisms in which both doxorubicin and healthy aging can influence heart function. Doxorubicin induces DNA damage, but also affects epigenetics and telomere length. In the cytoplasm, doxorubicin inhibits reuptake of calcium, leading to an increased calcium concentration. In the mitochondria, doxorubicin can by itself cause increased ROS production. Besides, it inhibits the electron transport chain, which leads to additional ROS production. In the nucleus chromosomes are affected by time as well. DNA damage occurs, epigenetics are altered, and telomeres become shorter. In the cytoplasm, reuptake of calcium becomes less efficient, leading to increased calcium concentrations. Age also affects the mitochondria so that the electron transport chain becomes less efficient, and more ROS is produced. All these mechanisms lead to similar outputs in the form of hypertrophy, diastolic dysfunction, increased incidence of atrial fibrillation, prolonged QT interval, and fibrosis.

The aging of the human body is accompanied by increasing numbers of senescent cells. Increased senescent cells are found in DCT models as well, for example in a transgenic model that permits the identification of cells expressing p16, a regulator of senescence^64^. Various cell types in the heart have been shown to become senescent upon doxorubicin treatment, including cardiomyocytes^65^, endothelial cells^66^, cardiac fibroblasts^61^, and cardiac progenitor cells^67^. While our primary focus is on cardiomyocytes, many of the processes described here are also applicable to the other cell types (Fig. 1)^65^.

### DNA damage and double-stranded break repair

DNA is a very stable molecule: intact DNA molecules of 700,000 years old have been recovered^68^. However, it is common knowledge that DNA damage arises on a day-to-day basis in several forms, including formation of oxidized nucleotides, single strand breaks, double strand breaks, and inter-strand crosslinks. The cell protects itself from the cytotoxic effects, by activating DNA damage response (DDR), which also consists of a range of mechanisms. These include base excision repair, non-homologous end-joining, and homologous recombination. Which of these is used depends on the type of damage and the speed and accuracy at which the DNA needs to be repaired. All these levels of protection cannot prevent accumulation of permanent mutations over time^69^. Doxorubicin also accelerates the DNA mutation rate. by interacting with DNA in two ways: by intercalating with the strands^70–72^, or by inhibiting TOP2b as mentioned above^73–75^. When doxorubicin intercalates with the DNA, it preferably binds to areas rich in GC basepairs^76^. The complex that is formed leads to supercoiling of the DNA strands, which results in increased stresses and the unwrapping of the DNA from the nucleosome^77^. The relevance of this mechanism in patients can be questioned, since only 4–5 adducts form every 10^7^ base pairs at clinically relevant doses^76^. There is more evidence for the activity of the second mechanism within patients, inhibition of TOP2b. Doxorubicin can influence TOP2b in two ways: it can prevent the protein from binding to the DNA or it can inhibit the final ligation step^74,78,79^. Inhibition of binding is due to the intercalation of doxorubicin with the DNA, but this does not happen in relevant frequency at clinical doses^74^. Thus, the second mechanism is a more likely candidate. When TOP2b is knocked out both in vitro^73,74^ and in vivo^75^, DCT can be prevented^74–76^. Additional confirmation of role of TOP2b in DCT comes from the use of dexrazoxane. Dexrazoxane administration simultaneously with doxorubicin has shown reduction of DCT incidence. Initially, it was believed that dexrazoxane mitigated DCT through iron chelation, but recent research indicates that it depletes TOP2a and TOP2b from tissues, thereby protecting the heart from toxicity^67,80^.

**Fig. 2:**
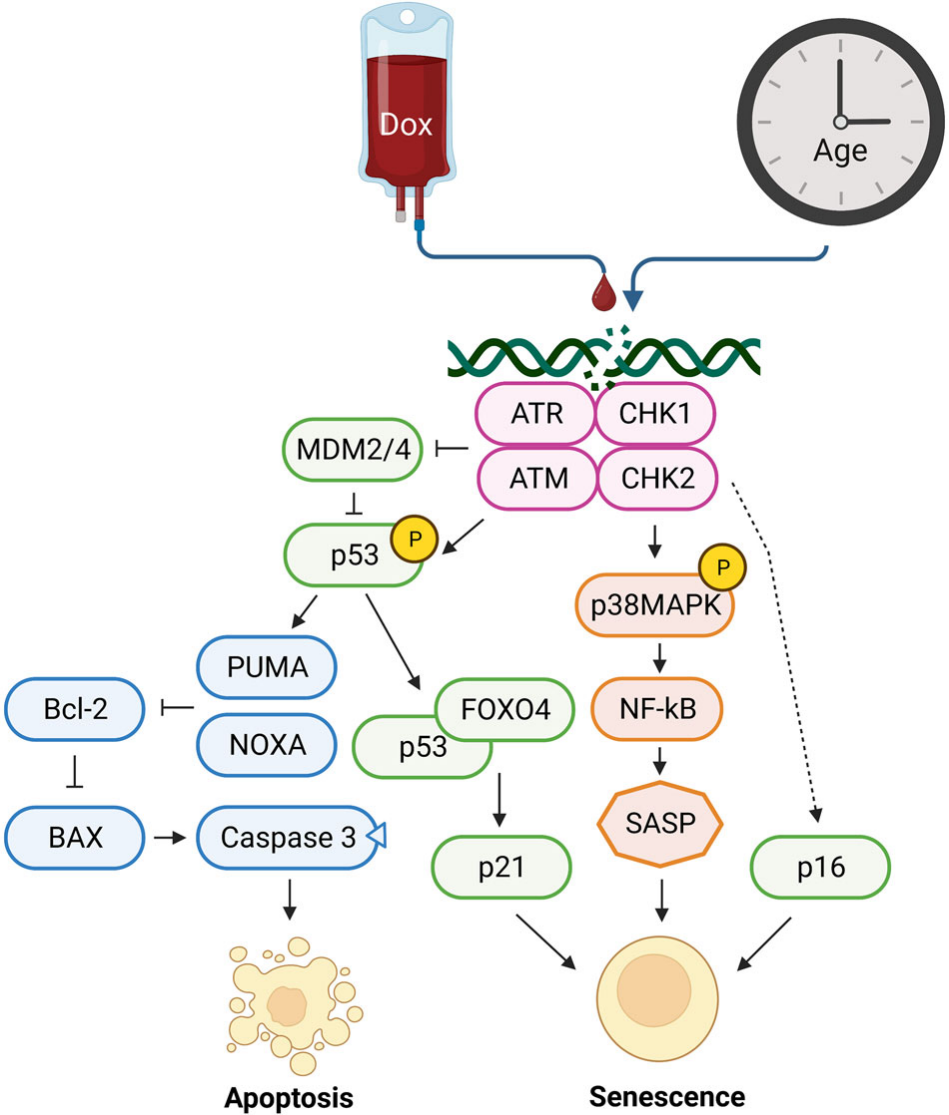
Pathways involved in DNA damage. After induction of DNA damage, ATR-CHK1 and ATM-CHK2 are recruited to the damaged position. These lead to an activation and increased presence of p53, by inhibiting MDM2/4. Activated p53 in turn either binds to FOXO4, inducing increased expression of p21, or interacts with PUMA and NOXA, which activates the apoptosis cascade. Besides activation of p53, ATR-CHK1 and ATM-CHK2 are also responsible for phosphorylation of p38MAPK and transcription of p16. These two proteins activate senescence in the cell.

DNA damage activates senescence-related pathways. The first proteins that react to DNA damage are Ataxia Telangiectasia Mutated (ATM), Ataxia Telangiectasia and Rad3-Related Protein (ATR), and Checkpoint Kinase 1 (Chk1) and 2 (Chk2). ATM-Chk2 is activated by double stranded breaks, while ATR-Chk1 reacts to single stranded breaks^81^. Inhibition of the cell cycle is accomplished through p53. In the healthy cell, p53 is a short-lived protein, which is rapidly ubiquitinated by Mouse Double Minute 2 homolog (MDM2) and MDM4 and degraded. ATM-Chk2 and ATR-Chk1 affect p53 in multiple ways. First, they stabilize it by inhibiting MDM2 and MDM4. Secondly, they also phosphorylate p53^82^. Once phosphorylated, p53 can either activate p53 upregulated modulator of apoptosis (PUMA) and NOXA, which are apoptosis regulators, or p21, which causes cellular senescence^69^. How it is determined which pathway is activated is not fully understood^82^, but Forkhead box protein O4 (FOXO4) is believed to be involved. Disruption of p53-FOXO4 complexes with a peptide causes senescent cells to go into apoptosis^83^. Another important senescence marker that activates upon DNA stress, independently from p53, is p16. Although postnatal cardiomyocytes are in cell cycle arrest at the G1/S restriction point, they show upregulation of these pathways regardless^14,16,18^ (Fig. 2).

Another regulator of senescence is p38MAPK. It can be activated by DNA damage, oxidative stress, ER stress, metabolic stress, and inflammatory stress. When activated, it can drive many other pathways, including p53, p21 (independent of p53), p16, and NF-kB pathways^84^. Treatment with doxorubicin has been shown to activate p38MAPK^14,85^.

### Telomeres

In addition to DNA damage, aging correlates with telomere shortening. To replicate DNA, DNA polymerase needs an RNA primer as a starting position. At the ends of the DNA strands, this results in the loss of basepairs with each replication cycle (the end replication problem). To protect the genetic material, the ends of chromosomes consist of telomeres. These are repeats of a random sequence, packaged in protein complexes known as shelterin. Shelterin prevents the recognition of chromosome ends as DNA damage^45^. With every replication, the telomeres become shorter, until they reach a critical length that disrupts the shelterin complex. This activates a sustained DDR and the cell stops proliferating. Telomerase can lengthen the telomeres again, but it is not expressed in all celltypes^86^. In a study of 530 autopsy subjects, it was shown that telomeres in the heart shorten at a rate of approximately 20 basepairs per year^87^. With age, an increase in DDR proteins was observed in the telomeres, independent from shortening^45,88^. In doxorubicin-treated human^18^ and mouse^16^ hearts, telomeres were shorter compared to controls. Telomere binding proteins^89^ and telomerase^16^ were also shown to be dysregulated in doxorubicin-treated hearts^16^.

### Epigenetics

DNA in the nucleus is wrapped around proteins (histones) and organized into chromosomes. Modification of packaging can control gene expression: tightly packed DNA cannot be transcribed (heterochromatin), while loosely organized DNA is available for transcription (euchromatin). The tightness of packaging is adjusted by modifications of the histones and of the DNA itself. Acetylation promotes formation of euchromatin, while methylation promotes heterochromatin^90^. Aging reduces histone levels and when they are experimentally elevated, lifespan is increased^91^. Doxorubicin treatment causes nucleosome turnover around the promoters of active genes^77^ and histone eviction and degradation^92^, resulting in decreased cellular histone levels.

DNA bases can be methylated, mostly in CpG islands: stretches of DNA less than 200 basepairs long, which contain >50% C-G pairs. CpG islands are often located in proximity to promotors. More methylated bases results in decreased gene expression^93^. Aging leads to an overall decrease of DNA methylation^93^. Doxorubicin decreases expression of DNA methyltransferases^94^, and methylation levels^95,96^. Methylation levels can be used to determine biological age, which has been linked to cardiovascular health^96–98^. Although DNA methylation in doxorubicin-treated models has been measured, biological age has not been assessed.

Epigenetic regulation ties gene expression to metabolism. During aging and doxorubicin treatment availability of substrates such as ATP, NAD^+^, and acetyl co-enzyme declines, resulting in heterochromatin^95,99^. Sirtuins, NAD^+^ dependent histone deacetylases, are regulators of this process. When there is less energy, more NAD^+^ is available, and Sirtiuns deacetylate histones, leading to heterochromatin^100^. The involvement of these proteins in aging and DCT has been studied extensively. Overexpression of several Sirtuins increased lifespan, while knockout decreased lifespan^100,101^. Several studies showed increased activity of Sirtuins protects the heart from DCT^51,85,102–104^.

### Mitochondrial dysfunction and mtDNA damage

Due to high energy demand, 40% of the cardiomyocyte’s cytoplasm is occupied by mitochondria^105^, Mitochondrial dysfunction, a primary characteristic of senescence, therefore plays a key role in cardiomyocytes. Improving mitochondrial function can suppress senescence^65^. Mitochondria have an inner (IMM) and an outer (OMM) mitochondrial membrane. In the IMM, the electron transport chain (ETC) is present consisting of four complexes (complex I-IV) that use NADH and oxygen to increase the proton content in the intermembrane compartment. ATP synthase (complex V) uses this gradient to phosphorylate ADP into ATP (oxidative phosphorylation). The IMM is folded into cristae, to increase the surface area available for oxidative phosphorylation^106^. During aging, organization of these cristae is disrupted^107,108^ and the activity of the ETC is decreased^106,109^. Both are a result of changing phospholipid content of the IMM and the resulting decreased membrane fluidity. This disrupts the organization of the ETC complexes, interrupting efficient function^106^. Cardiolipin, a phospholipid exclusive to the IMM, is a likely candidate. Its conical structure enables the membrane to form curves^109^. Cardiolipin content decreases with age and experimental addition of it to aged mitochondria increased function^109,110^. Doxorubicin accumulates in mitochondria, partially by binding to cardiolipin^111,112^. The phospholipid has an anionic charge, while doxorubicin has a cationic charge, resulting in the formation of acomplex^9,113^. Similar to aged-induced dysfunction, this bond disrupts the organization of the cristae^104,114–116^ and inhibits proper function of ETC^117^. Other processes that are affected in aged cells include a shift to glycolysis, increased proton leak, decreased membrane potential, and increased ROS production^58,108,118,119^. These changes also take place in doxorubicin-treated cells^51,53,54,95,104,114,116,120–124^. In senescent fibroblasts, mitochondrial function decreased, possibly due to impaired mitophagy^125^. Doxorubicin-treated fibroblasts show a similar phenotype, possibly mediated by p53^61^. Mitochondria have small circular mitochondrial DNA (mtDNA) strands. mtDNA encodes a few proteins, which all are involved in ATP-production. There are less repair mechanisms in place for mtDNA, therefore the mutation rate is 10–17 times higher than in nuclear DNA^95^. Over time, mutation in the mtDNA accumulate, resulting in mitochondrial dysfunction^106^. The role of mtDNA in aging was confirmed in a mouse-model expressing a defective mitochondrial DNA polymerase: it showed an accelerated aging phenotype^119,126^. Doxorubicin is a molecule that can interact with DNA. It increases mutation rate and dysfunction of mitochondria^95,104^.

### Reactive oxygen species and oxidative stress

Mitochondria are a source of free radicals such as ROS. In a healthy cell, there is a balance between the production of free radicals and the activity of antioxidant enzymes and molecules. However, when this balance is disrupted, the cell will enter a state of oxidative stress^65^. The heart is particularly vulnerable to oxidative stress, due to high metabolic activity, production of radicals for signaling purposes, and low amounts of antioxidants and antioxidant enzymes^19,117,127^. The presence of free radicals causes sulfhydryl oxidation, lipid peroxidation, cardiolipin reduction, and mtDNA damage in the mitochondria. This leads to a further increase in free radical production. Free radicals can cross over to organelles that are in close proximity, such as the endoplasmatic reticulum. The cell will respond by uncoupling the oxidative phosphorylation from ATP production (proton leak), increasing activity of the ETC complexes^106,117^. It has been shown that both aging processes and doxorubicin treatment increases ROS production in cardiomyocytes^19,128^ and cardiac fibroblasts^61,129^.

During aging, the activity of the ETC complexes decreases. This increases ROS molecule production^106^. In doxorubicin-treated cells, activity of the ETC also decreases with similar results. Besides that, doxorubicin is a quinone molecule which can be reduced by several enzymes, including cytosolic xanthine oxidase, NADH dehydrogenase (complex I), and NADPH-dependent cytochrome P450 reductases. After reduction, it auto-oxidizes to its neutral state. During this second step, a superoxide anion is generated^130,131^. Doxorubicin also reduces expression of antioxidant enzymes^65,132^.

Overall, it can be concluded that ROS production increases in both aging cells and in doxorubicin-treated cells. In recent years, several studies suggest that increasing ROS may not be the causative factor of both aging^19^ and DCT^133^. While suppression of ROS production is beneficial in aged animal models^134^, in the healthy aged population suppression of ROS production decreased lifespan in some cases, possibly due to the signaling function some of these molecules have^135–137^. With doxorubicin treatment, similar objections have been suggested. Firstly, free radicals do not form immediately after treatment^19^. Antioxidant enzymes could not prevent doxorubicin-induced DNA damage, indicating another mechanism^74^. Secondly, clinical trials showed that antioxidants could not prevent the accelerated aging phenotype in DCT patients^19,138,139^, while in animals upregulation of antioxidant enzymes has a beneficial effect^48,127^. Lastly, it remains unclear whether antioxidant therapy influences the cancer efficacy of doxorubicin, rendering it impractical for clinical use^140^.

### Calcium flux

Calcium regulates contraction of cardiomyocytes. During an action potential, Ryanodine Receptor 2 (RyR2) releases calcium from the sarcoplasmic reticulum (SR). This calcium spike activates the sarcomeres, and the cell shortens. Subsequently, SR Ca^2+^-stimulated ATPase (SERCA) pumps calcium back into the SR and the cell relaxes. In both aged and doxorubicin-treated mice the amount of phosphorylated troponin I increased, indicating that relaxation of the cardiomyocyte is impaired^16^. In human aged cells, calcium concentration remains high for longer and relaxation is slower^44,141^. RyR2 and SERCA are responsible for these changes. The likelihood of RyR2 being in its open conformation increases with age (leaking), while it is activated by doxorubicin treatment^142–144^. SERCA activity has been shown to be decreased both with age and doxorubicin treatment^123,144^. This is partially due to decreased phosphorylation of phospholamban, an inhibitor of SERCA, and decreased ATP content^123^.

## Involvement of the immune system in aging and DCT

The immune system’s response to aging and DCT illustrates a complex interplay, characterized by the secretion of chemokines, cytokines, and growth factors, collectively forming the senescence-associated secretory phenotype (SASP). During aging, the upregulation of pro-inflammatory cytokines such as IL-6 and TNF-α, alongside chemokines like CCL2 and CXCL12, establishes a chronic inflammatory state within the heart^145,146^. This inflammatory environment induces cellular stress and fosters cardiac cell senescence, contributing to the decline in cardiac function.

In the context of DCT, a similar pro-inflammatory response is demonstrated, marked by the elevation of SASP factors. Studies have demonstrated that expression of cytokines such as IFN-γ, CCL27, and MIF is increased in patients undergoing doxorubicin treatment, linking them to the DCT process^64,147–149^. This response is part of a broader systemic reaction, where the immune system attempts to mitigate the damage caused by the chemotherapeutic agent but instead contributes to the deterioration of cardiac tissue.

The role of growth factors within the SASP, such as TGF-β and PDGF, is critically involved in cardiac remodeling and fibrosis, processes that are prominent in both aging and DCT^64,150^. The presence of increased SASP production both in DCT and aging underscores the concept of an accelerated aging phenotype in DCT. This similarity in cytokine, chemokine, and growth factor expression and activity between aging and DCT suggests shared mechanisms, offering potential therapeutic targets or biomarkers^151,152^. Modulating the inflammatory response could present new strategies for mitigating the effects of both aging and DCT. For example, Zymosan A has been shown to improve cardiac healing and ameliorate doxorubicin-induced ventricular remodeling. This was achieved by a heightened cardiac inflammatory response, leading to enhanced repair in DCT mice^153–155^.

## Cardiac senescence in clinical practice

### Senescence as a biomarker

Senescence could be used as a biomarker for predicting outcomes of DCT. Senescent cells secrete SASP factors, which can be detected in the blood. Several SASP factors have already been linked to worse prognosis in cardiovascular disease. Examples are IGFBP7^156,157^, interleukin-6 (IL-6)^158,159^, and GDF-15^65,160^. Patients with chronic heart failure (BIOSTAT-CHF) with elevated levels of IGFBP7 showed a 44% (HR = 1.44 [CI 1.23–1.70], *p* < 0.001) increase in combined adverse endpoints^156^. Increased IL-6 levels have also been reported in women who experienced cardiovascular events versus healthy women (1.65 versus 1.30 pg/ml, *p* = 0.003)^159^. GDF-15 is upregulated upon doxorubicin treatment^14^ and has been proposed as a cardiomyocyte-specific SASP factor^65^. Higher levels of GDF-15 in the blood predict worse outcome: A significant correlation between GDF-15 and death (HR = 1.66 [SD 1.07–1.26] *p* < 0.001), heart failure (HR = 1.52 [SD 1.29–1.78], *p* < 0.001), and major cardiovascular events (HR = 1.26 [SD 1.14–1.41], *p* < 0.001) was found^160^. However, large trials investigating SASP levels in the blood of patients treated with doxorubicin have not been conducted.

### Senotherapeutics

Research has indicated that a promising approach to protect the heart against DCT involves the removal of senescent cells. By employing a transgenic model that enables the targeted elimination of p16-expressing cells, cardiac function was successfully restored^64^. Consequently, two classes of drugs, known as senolytics and senomorphics, hold potential for both DCT treatment and the broader context of combating aging. Senolytics cause cell death of senescent cells specifically, while senomorphics prevent cells from becoming senescent by modulating SASP expression.

Senescent cells can resist apoptosis by upregulating anti-apoptotic genes such as Bcl-2^129^. Senolytics target this phenotype to remove senescent cells from a tissue. This has beneficial effects on health-span in general^161^. The first senolytic compounds to be discovered, were dasatinib and quercetin. When administered together, heart function in 24-month-old mice was improved^162^, but they were never tested in DCT. Another compound that was designed to inhibit anti-apoptotic proteins is navitoclax^163^. Navitoclax is being investigated in clinical trials as an anti-tumor drug for multiple cancer types and the results are promising^164^. It has also been shown to improve cardiac function in doxorubicin-treated mice^165^. FOXO4-DRI is a senolytic that was designed to disrupt p53-FOXO4 complexes, causing p53 to initiate apoptosis instead of senescence^83^. This peptide can protect against doxorubicin-induced liver damage, but its effect on DCT has not been investigated^83^. Fisetin is a bioflavonoid that can be found in many types of plants and fruits, including strawberries and apples. It has senolytic properties and increases healthspan^166^. In doxorubicin-treated rats, it has a beneficial effect on heart function^167^.

Senomorphics include a wide range of compounds, many of them natural products. They affect the cells in multiple ways, making their exact mechanism of action difficult to establish^161,168^. Several have been shown to be effective against DCT in animal models: resveratrol^169^, metformin^170^, PJ34^171^, and 5-aminoimidazole-4-carboxamide ribonucleotide (AICAR)^172^. For example, rats that were co-treated with doxorubicin and AICAR had a preserved ejection fraction after six weeks^172^. Human data on the efficacy of these compounds in DCT is scarce and mostly based on in vitro research^173–175^. The effect of resveratrol in combination with other compounds on cardiovascular disease has been investigated. The results of these trials were marginally positive, but the effect of resveratrol alone was not assessed^173^. Besides, conventional heart failure therapies have limited efficacy in DCT patients^176–178^. Therefore, the effect of resveratrol on DCT could differ from what is observed in other cardiovascular etiologies. This was supported by a recent study from our lab. iPSC-CM-based dynamic engineered heart tissues were subjected to four clinically relevant doses of doxorubicin over the course of four weeks and co-treated either with resveratrol or AICAR. Neither resveratrol nor AICAR was able to improve the phenotype induced by doxorubicin. It was suggested that prevention of senescence resulted in apoptosis and ultimately in worsening of the function in the case of the AICAR treated tissues^14^. This indicates that treatment with senomorphics in DCT could be detrimental and that preclinical experiments using animals might not be accurate predictors of the outcome in humans.

## Future perspectives

Further research into the underlying mechanisms linking cardiovascular disease and cancer is one of the Gaps in Evidence highlighted by the ESC 2022 Guidelines on Cardio-Oncology. In this review, we have discussed the evidence of an accelerated aging phenotype in the hearts of DCT patients. A comparison was made between the healthy aged heart and the doxorubicin-treated heart. On a macroscopic level, there were some similarities, such as hypertrophy and fibrosis. Most resemblances were found at cell level, where we described an increased number of mutations, upregulation of senescence markers, shorter telomeres, declining histone levels, decreased DNA methylation, decreased Sirtuin activity, less availability of substrates for epigenetic modifications, decreased energy production, increased mtDNA damage, increased ROS production, and dysfunctional calcium handling. Doxorubicin was in all instances able to initiate mechanisms that would otherwise happen gradually with time, suggesting doxorubicin treatment leads to an accelerated aging phenotype. Although this review discusses most well-known processes involved in both aging and doxorubicin treatment, not all are elaborately explained. Mechanisms for which less evidence exists, or for which evidence is controversial were omitted, for example autophagy^49,179,180^, and inflammation^45,59^.

More research on the involvement of senescence in DCT is merited. However, special care should be taken in selecting the experimental model. Rats and mice are much more resilient to doxorubicin than humans, meaning that higher dosages of doxorubicin have to be administered. It is known that different concentrations of doxorubicin activate separate mechanisms, suggesting that DCT in mice and rats might have a different origin than in humans. Besides that, we have seen that the effect of senolytics is positive in mice and rats with DCT, while it worsens function in human 3D models. Parallel to this, it has been observed that antioxidant molecules have a beneficial effect on experimental animal models of DCT and not in clinical trials. This discrepancy could be due a difference in underlying mechanism, or to higher regeneration rates of cardiomyocytes in mice (1.3–4%^181^) compared to humans (0.3–1%^182^).

All this knowledge is instrumental in developing cardioprotective strategies. Numerous avenues are being explored to protect the heart from DCT, including the use of dexrazoxane^80^, honokiol^104^, and curcumin^183^, many of which target either mitochondrial function or inflammation. Another cardioprotective strategy involves co-treating with doxorubicin and SGLT-2 inhibitors. A retrospective study including 32 patients who had received doxorubicin and SGLT-2 inhibitors showed no development of DCT^184^. In a pre-clinical model of DCT, empagliflozin showed decreased fibrosis, decreased inflammation, decreased oxidative stress, decreased lipid peroxidation, and increased mitochondrial biogenesis and left ventricular function^185^.

All this evidence taken together suggests that the heart undergoes an accelerated aging phenotype after doxorubicin exposure. Since many differences between the human heart and the heart of animals are observed, this should be taken into account when designing new experiments.
